# Recent Differentiation of Aquatic Bacterial Communities in a Hydrological System in the Cuatro Ciénegas Basin, After a Natural Perturbation

**DOI:** 10.3389/fmicb.2022.825167

**Published:** 2022-04-28

**Authors:** Manuel García-Ulloa, Valeria Souza, Diego A. Esquivel-Hernández, Jazmín Sánchez-Pérez, Laura Espinosa-Asuar, Mariette Viladomat, Montserrat Marroquín-Rodríguez, Marisol Navarro-Miranda, Jair Ruiz-Padilla, Camila Monroy-Guzmán, David Madrigal-Trejo, Manuel Rosas-Barrera, Mirna Vázquez-Rosas-Landa, Luis E. Eguiarte

**Affiliations:** ^1^Departamento de Ecología Evolutiva, Instituto de Ecología, Universidad Nacional Autónoma de México, Ciudad de México, Mexico; ^2^Departament de Genètica i Microbiologia, Facultat de Biociències, Universitat Autònoma de Barcelona, Barcelona, Spain; ^3^Centro de Estudios del Cuaternario de Fuego-Patagonia y Antártica (CEQUA), Punta Arenas, Chile; ^4^Facultad de Medicina, Licenciatura en Investigación Biomédica Básica, Universidad Nacional Autónoma de México, Ciudad de México, Mexico

**Keywords:** Eltonian niche, Grinnellian niche, founder effect, environmental filtering, compositional differentiation, ecological perturbation

## Abstract

Pozas Rojas is a hydrological system comprising nine isolated shallow ponds and a deep lagoon, which were temporally merged in 2010 by increased rainfall due to a tropical cyclone. In this work, we assess which components, biotic interactions, or environment filtering effects, drive the assembly of microbial communities after a natural perturbation. Arsenic, pH, and temperature are among the most significant environmental variables between each pond, clustering the samples in two main groups, whereas microbial composition is diverse and unique to each site, with no core at the operational taxonomic unit level and only 150 core genera when studied at the genus level. Los Hundidos lagoon has the most differentiated community, which is highly similar to the epipelagic Mediterranean Sea communities. On the other hand, the shallow ponds at the Pozas Rojas system resemble more to epicontinental hydrological systems, such as some cold rivers of the world and the phreatic mantle from Iowa. Overall, despite being a sole of water body 2 years prior to the sampling, interspecific interactions, rather than environmental selection, seem to play a more important role in Pozas Rojas, bolstered by founder effects on each poza and subsequent isolation of each water body.

## Introduction

Early in the 20th century, two main frameworks for the study of ecological niches have been proposed: the Grinnellian and Eltonian niche concepts. On one hand, the Grinnellian niche concept (Grinnell, 1904) was meant as a description of the complete range of conditions and resources where any given organism could live and reproduce (see review in Soberón, 2007). On the other hand, the Eltonian (Elton, 1927) niche concept is explained by the interactions among species, with the trophic web as central for the establishment of local community assembly (Soberón, 2007). Although there are multiple studies on the interplay of the Grinnellian and Eltonian niches in natural animal and plant populations (Soberón, 2007; Larson et al., 2010; Rosado et al., 2016; Junker et al., 2019), their role in succession processes and perturbation scenarios is more difficult to dissect, and questions regarding the relative importance of local environmental conditions and interactions among members of the community remain to be answered, particularly in *in situ* microbial setups.

Succession process in aquatic microbial communities is seldom studied *in situ* given the complexities of natural communities and a large amount of confounding factors, including community response to environmental variables and stressors, stochastic processes, migration, and founders’ effect (Pajares et al., 2013, 2015; Pascual-García and Bell, 2020). However, aquatic mesocosm experiments have shown that environmental variables such as temperature and UV light determine the response of the microbial community to perturbation, as the Grinnellian niche concept would predict. Other studies have shown the influence of the Eltonian niche. For instance, the establishment of bacterial communities in beech tree holes seems to be governed by functional redundancy and founders’ effect (Pascual-García and Bell, 2020). In contrast, communities from oligotrophic lakes in the Pyrenees have shown to be influenced by the interactions between environment and the community as well as migration processes, being environmental filtering a strong force (Ortiz-Álvarez et al., 2020). Therefore, both the Grinnellian and Eltonian niches are relevant for the establishment of the lake microbial communities.

Another fundamental issue is what happens to microbial communities after natural perturbations, that is, when the Grinnellian niche changes abruptly. In a 4-year study of halite communities from the Atacama Desert, after an unusual rain, the community shifted to a completely new unstable state and then recovered through stochastic recolonization once the water evaporated (Uritskiy et al., 2019). Opposite to the Atacama example, in the Churince system from Cuatro Ciénegas Basin (CCB), water became scarce, and salinity progressively rose due to the overexploitation of the aquifer. This resulted in demographic fluctuations of the *Pseudomonas* community, producing a sudden bloom of *Pseudomonas aeruginosa* (García-Ulloa et al., 2019) and an event of evolutionary rescue of *Pseudomonas otitidis* (García-Ulloa et al., 2021). However, as the perturbation became stronger and disrupted the community, the entire genus disappeared and a never-before-seen environmental strain of *Vibrio cholerae* appeared close to the extinction of the lagoon. The interplay between Grinnellian and Eltonian niches seems to explain this outcome; as the Grinnellian niche radically changed, the Eltonian niche eventually became altered, and the original community collapsed. Unfortunately, as in the case of *P. otitidis* and possibly most of the *Pseudomonas* genus, the enormous diversity of Churince [5,167 operational taxonomic units (OTUs) in a square km (Souza et al., 2018)] is now mostly lost due to the water depletion.

Within the studied sites at CCB is Pozas Rojas, located on the east side of the valley and composed of multiple shallow pozas and a deeper lagoon. This place was studied in parallel to Churince as a comparative site for cultivated *Bacillus*, *Exiguobacterium*, and *Pseudomonas* (Avitia et al., 2014) and more recently for *Vibrio* (Vázquez-Rosas-Landa et al., 2017, 2020). It is interesting to note that earlier metagenomics of Pozas Rojas observed a strong dominance and diversity of *Pseudomonas*, a lineage that probably benefited from the environmental filtering due to the extreme unbalanced stoichiometry of the site (C:N:P 15,820:157:1; Bonilla-Rosso et al., 2012; Peimbert et al., 2012). However, the extreme oligotrophy of Pozas Rojas was perturbed by Hurricane Alex in April 2010. This natural perturbation brought not only water that temporarily connected all pozas and the lagoon into one body of water, but also debris from most of the east side of the basin to the region named *Los Hundidos* due to its karstic nature and slightly lower elevation than the rest of the basin. As time passed, the superficial excess of water refilled the deep aquifer, leaving in 2012 the pozas in the same geographical position they were before the perturbation. At the moment of the sampling in 2013, the site was no longer oligotrophic (C:N:P 350:9:1) allowing the more copiotroph *Vibrio* to thrive (Vázquez-Rosas-Landa et al., 2020). Also, in a recent sampling of Pozas Rojas viral diversity, compared with Churince, the pozas showed a much larger diversity and differentiation in the water column between water bodies in March 2014 (Taboada et al., 2018), a year after the present study.

Herein we studied the aquatic microbial diversity of Pozas Rojas, 3 years after Hurricane Alex merged all the pozas into one lake and then got reseparated. In this work, we want to address the issue of differentiation between pozas in a very short time, testing the hypothesis of Grinnellian versus Eltonian niche as drivers of the community structure. As a null hypothesis, the flooding erased the structure between pozas within Pozas Rojas, and at that time, they showed no significant differentiation. However, there are two alternative hypotheses: (1) the Grinnellian niche drives the pozas differentiation; therefore, environmental variables explain most of Pozas Rojas diversity; (2) each poza will be unique within Pozas Rojas due to the Eltonian niche where interactions rule community assembly. Moreover, we scale such geographical differentiation from the local to a more global scale by comparing the Pozas Rojas composition with other epicontinental and marine water bodies: the phreatic mantle from Iowa, a groundwater system; the Mediterranean Sea, a marine system; several cold rivers from the world, which are instances of lotic systems; the Churince system from the CCB; and the well-studied Lake St. Clair in Michigan, a lentic system that is part of the Great Lakes Basin. We expected sites within Pozas Rojas to be more similar among each other than other CCB sites, such as Churince. Following this logic, CCB’s systems would be more similar between them than between different sites of the world, as Pozas Rojas and Churince may share a core of CCB microbiota given their shared deep aquifer (Wolaver et al., 2013).

## Materials and Methods

### Sample Collection, DNA Purification, and Sequencing

Pozas Rojas system, within the CCB, is located at the geographic coordinates 26°52′N, 102°1′W. Sampling site overview and geographic coordinates for each poza/lagoon are provided in the Supplementary Figure 1 and Supplementary Table 1. The pozas (designated S02–S09 in the present study) are small and shallow (no more than 20 cm in the winter); therefore, wind is expected to evenly mix the water column, except at the Los Hundidos (designated LH1) lagoon, which is roughly 10 m deep, and stratification is presumed. Each water body was sampled with sterilized water bottles, collecting 6 L of superficial water and keeping them in a cold icebox. Simultaneously, environmental variables were measured with a Hydrolab MS5 multiprobe. Three liters from each water body was taken for mineral and nutrient determination (CIECO and Instituto de Geofísica, UNAM). Microbes were collected by filtering *in situ* all the sampled water onto sterile GF/F filters (0.2-mm nominal pore size; Whatman, Piscataway, NJ, United States) using a Millipore filtering device. GF/F filters were used in order to accumulate enough biomass for DNA extraction.

DNA was extracted from water column samples using the MOBIO PowerWater DNA Isolation kit (MoBio Laboratories, Carlsbad, CA, United States), with one modification: the volume of PW1 solution was increased to 1.5 mL due to the high absorbency of the GF/F filters, obtaining several filters per poza. Therefore, a composed sample of several independent DNA isolation steps from each site was pooled into one sample per poza. The DNA was used to amplify the V4–V6 16S rRNA gene variable regions using the primers 357F and CD[R] (Rudi et al., 1997; Turner et al., 1999). Amplicon libraries (450–490-bp length) were constructed as reported in the 16S Metagenomic Sequencing Library Preparation protocol from Illumina and sequenced on the Illumina MiSeq platform with a paired-end read configuration of 150 cycles at CINVESTAV-LANGEBIO, Irapuato, Mexico.

### Measurement of Physicochemical Parameters

To determine the ionic concentration of the samples, sediment samples were incubated with distilled water for 19 h at 25°C under continuous shaking. This procedure allows the mobilization of the available ions within the sediment. Liquid samples from sediment and water were filtered with 0.22 nitrocellulose membranes. Concentrations of CO_3_^2–^ and HCO_3_^–^ anions were determined by HCl 0.01 N titration. Cl^–^ and SO_4_^2–^ concentrations were determined by high-performance liquid chromatography. A 432-conductivity detector (Waters) and an IC-Pack HR-Waters column were used. The mobile phase was a mixture of sodium borate (1.3 M) and acetonitrile (12%) at pH 8.5. Ca^2+^ and Mg^2+^ concentrations were obtained by atomic absorption spectroscopy using a Perkin Elmer 3110 equipment. Na^+^ and K^+^ were determined by flamometry using a Corning 400 device. For nutrient quantification, sediment samples were dried, and water samples were filtered through a Millipore 0.42-μm filter. Total carbon (TC) and inorganic carbon (IC) were determined by combustion and colorimetric detection using a TC analyzer (UIC model CM5012, Chicago, IL, United States). Total organic carbon was calculated as the difference between TC and IC. For total N and total P (TP) determination, samples were acid digested with H_2_SO_4_, H_2_O_2_, K_2_SO_4_, and CuSO_4_ at 360°C. Soil N was determined by the macro-Kjeldahl method (Bradstreet, 1954), whereas P was determined by the molybdate colorimetric method following ascorbic acid reduction (Dick and Tabatabai, 1977). The N and P forms analyzed were determined colorimetrically in a Bran–Luebbe Autoanalyzer 3 (Norderstedt, Germany). Several replicates per measurement were used in the statistical analysis.

### Microbial Diversity

A total of 223,937 reads were obtained with MiSeq Illumina 2 × 300 using 357F and 939R primers. Reads passing the QC filters (minimum read quality = Q20) were used to reconstruct the original amplicon region (450–490-bp length) by overlapping them with Flash v1.2.7 software (Magoč and Salzberg, 2011). All non-overlapping sequences were discarded. A total of 188,190 sequences were used for taxonomic annotation with the Parallel-META v2.4.1 pipeline (Su et al., 2014) against the Metaxa2 database v2.1.1 (Bengtsson-Palme et al., 2015; Escobar-Zepeda et al., 2018). Stacked bar plots at different taxonomic levels were generated with ggplot2 (Wickham, 2016). The OTU abundance table was used to calculate the Good’s coverage (rarefaction) and α diversity indexes, that is, Observed, Chao 1, Fisher, Simpson, Inv Simpson, and Shannon, with the R Phyloseq library (McMurdie and Holmes, 2013). The OTU table was normalized using the metagenomeSeq method (Paulson et al., 2013), and the β diversity distance matrix was calculated using Bray–Curtis dissimilarity. Absence/presence plots were generated using the Upset R library (Lex et al., 2014) from the abundance OTU table and taxonomic annotation table at the genus level.

### Statistical Analyses

Statistical analyses were conducted with R.^1^ Using the physicochemical and environmental measurements, the optimal number of clusters was assessed, from *k* = 2 to *k* = 8, with cluster v2.1.0, and plotted with factoextra v1.0.7 R packages, respectively (Kaufman and Rousseeuw, 2009). Pearson correlation coefficients were calculated to assess the associations between microbial α diversity and environmental measurements and the relationships across various environmental variables. *p* < 0.05 was considered to be statistically significant.

Non-correlated variables were selected based on a correlation plot calculated with the R package corrplot v0.84 (Wei and Simko, 2017), resulting in eight independent environmental variables: temperature, C:N, calcium (Ca), iron (Fe), conductivity, TP, pH, and arsenic (As). However, Fe was excluded from the analysis because of its slight presence in only two of the samples. Euclidean dissimilarity matrices of individual and every possible group of non-correlated environmental variables and a Harvestine dissimilarity matrix of the coordinates from Pozas Rojas samples were calculated. Mantel tests were performed with both Spearman and Pearson correlation methods (9999 permutations) using the R package vegan v2.5.6 (Dixon, 2003). In addition, a canonical correspondence analysis (CCA) of the environmental and physicochemical measurements was performed using the cca function from the R package vegan v2.5-6. Only variables with *p* < 0.05 are shown to reduce noise.

### Data Acquisition of Worldwide Water Bodies and Taxonomic Annotation

Pozas Rojas raw reads were uploaded to MG-RAST and are publicly available on the site^2^ with the project accession number mgp94066. 16S tags from water samples collected globally were used as references. Accession numbers of the reference sites are as follows: mgp82906 (phreatic zone, Iowa), mgp10029 (eastern Mediterranean Sea), mgp89153 (Lake St. Clair, Michigan), mgp83892 (cold rivers, worldwide), and mgp94070 (Churince, Cuatro Cienegas, Mexico). All 16S tags used in this study were processed with the MG-RAST pipeline (Keegan et al., 2016). Rarefaction curves of the species count were performed for all geographical sites, and taxonomic annotation was done using the Ribosomal Database Project database as reference. An abundance table was constructed with the following minimum values: *e* = 10e−5, percentage of identity = 60%, length = 15 bp, abundance = 1.

### Comparative Analysis With Samples From Around the World

Bray–Curtis dissimilarity matrices of relative abundances and non-metric multidimensional scaling (NMDS) analyses of all taxonomic categories, from phylum to genus, for all sites around the world were calculated using the R package vegan v2.5.6. Homogeneity of multivariate dispersion at each taxonomic category was assessed with an analysis of variance (ANOVA) of the β dispersions of the Bray–Curtis dissimilarity matrices previously calculated. Validity of the NMDS analyses was assessed with the stress scores. A permutational multivariate ANOVA (1,000 permutations) using dissimilarity matrices and an analysis of similarity (ANOSIM) were carried out to measure significant differences between sites based on their taxonomic abundances with the adonis and anosim functions from vegan v2.5.6, respectively. In addition, a stacked bar plot of the relative abundances of phyla from all sites was done with the R package ggplot2. Rarefaction curves for each sample are shown in Supplementary Figure 4.

## Results

### As, pH, and Temperature Are the Most Important Environmental Variables in Pozas Rojas

**FIGURE 1:**
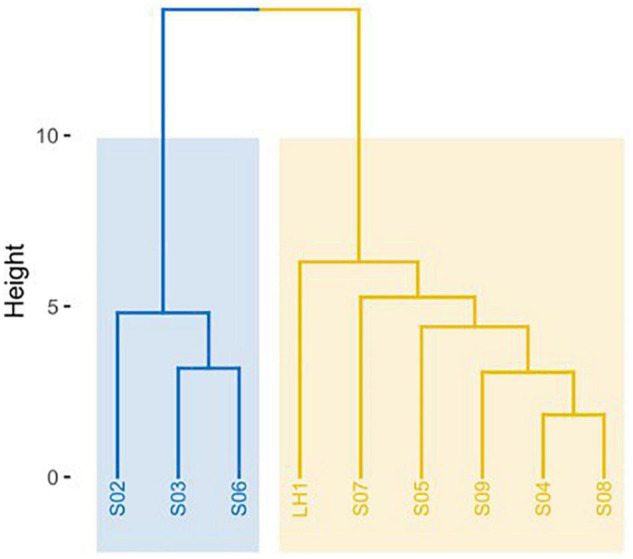
Dendrogram of environmental variables. Hierarchical *K*-means clustering algorithm of the R package cluster v2.1.0 was used. Two main groups resulted from the clustering of environmental variables, shown in blue and yellow.

Nutrient analysis showed that the proportion of C:N:P was, on average, 350:9:1 for water and 258:21:1 for sediment. These nutrients (C, N, and P) were not significantly different between sampling points for water samples (Vázquez-Rosas-Landa et al., 2020). Environmental variables include pH, temperature, conductivity, and the concentration of the following chemical species: CO_3_^2–^, HCO_3_^–^, SO_4_^2–^, Cl^–^, Na^+^, K^+^, Ca^2+^, Mg^2+^, Cd, Pb, and As (Supplementary Table 2). Pozas were separated by such environmental variables into two groups: LH1, S04, S05, S07, S08, and S09 in one group and S02, S03, and S06 in another (Figure 1).

Univariate Mantel tests show nine significant correlations of geographic location with temperature and As [As (Spearman): *r* = 0.615, *p* = 0.0164; temperature (Spearman): *r* = 0.366, *p* = 0.038; As (Pearson): *r* = 0.818, *p* = 0.0071; temperature (Pearson): *r* = 0.467, *p* = 0.013], and multivariable Mantel points to temperature and pH [temperature + pH (Spearman): *r* = 0.404, *p* = 0.037]. In addition, CCA gives significant results for pH and conductivity (Table 1).

**TABLE 1:** Canonical correspondence analysis of environmental variables.

	Data	χ ^2^	*F* stats	Pr (>*F*)	Significant variables
All	Temperature, pH, conductivity, C:N, C:P, N:P, COT, NT, PT, CO3, HCO3, SO4, Cl, Na, K, Ca, Mg, Cd, Pb, As	1.70192	1.0343	0.188	COT (*p* = 0.034)
Environmental	Temperature, pH, conductivity	1.10427	2.2101	0.013	pH (*p* = 0.001) Conductivity (*p* = 0.007)
Stoichiometry	C:N, C:P, N:P	0.97696	1.696	0.088	C:P (*p* = 0.004)
Minerals	COT, NT, PT, CO3, HCO3, SO4, Cl, Na, K, Ca, Mg, Cd, Pb, As	1.70192	1.0343	0.216	CO3 (*p* = 0.05)

### Each Poza Possesses a Distinctly High Microbial Diversity

**FIGURE 2:**
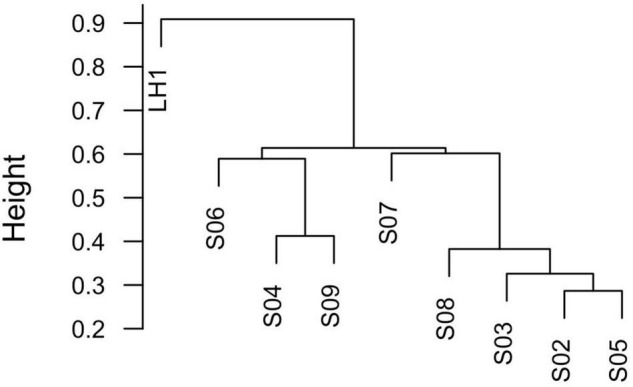
Dendrogram of Bray–Curtis distance between samples.

**FIGURE 3:**
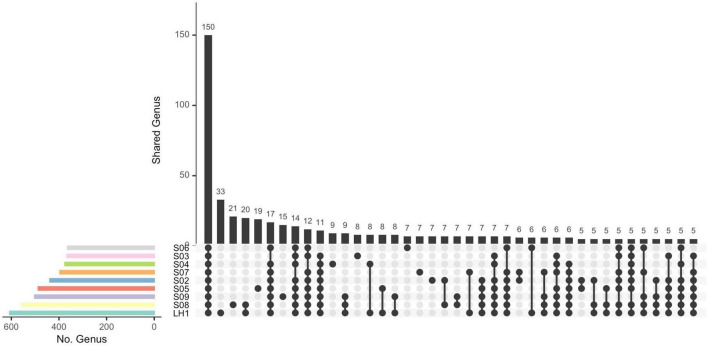
Upset plot of shared genera between pozas. Connected points represent the different combinations of sample intersections. First vertical frequency bar displays the common genera between all ponds. The total number of genera in each pond is shown by the horizontal frequency bars.

From a total of 188,190 reads, 5,268 OTUs were obtained at 97% similarity. Rarefaction curves show that even if the number of reads per poza is not the same, whereas LH is very well sampled along with S02, S08, and S09, the other pozas are subsampled (Supplementary Figure 2). However, microbial diversity is not related to the sequence coverage or to environmental variables. Community differentiation shows an isolated site (LH1) and two more related clusters (Figure 2 versus 1). The Los Hundidos site has clearly a unique microbial composition. This is confirmed by Bray–Curtis β diversity that is, in general, also very high, showing that each site is diverse and unique (Figures 2, 3 and Table 2). Furthermore, a Mantel test shows that this differentiation is unrelated to geographic distance (*p* = 0.2274, *R*^2^ = 0.03291), despite that the somewhat closer pozas (S02, S05, and S03) are more similar among each other in terms of community composition (Figure 2).

**TABLE 2:** Microbial α diversity in the water of the Pozas Rojas hydrological system after a natural perturbation.

Sample	Observed	Chao1	Shannon	ACE	Simpson	InvSimp	Fisher
LH1	1,851	1,880.57	4.12	17.7	0.92	12.24	365.59
S02	1,143	1,449.02	2.85	22.95	0.71	3.5	235.81
S03	415	893.85	4.54	19.91	0.96	27.5	161.68
S04	736	1,220.71	5.06	22.55	0.98	49.85	255.53
S05	1,271	1,734.97	4.92	25.37	0.97	29.97	365.65
S06	905	1,323.49	4.94	21.68	0.97	32.46	271.47
S07	497	664.2	3.04	16.63	0.72	3.55	137.69
S08	1,841	2,358.46	5.43	28.8	0.98	42.28	501.55
S09	1,123	1,448.9	4.28	23.36	0.95	20.21	290.76

Despite its recent recolonization, the diversity and equitativity within each site is very high as reflected by the high Shannon and Simpson indexes, with the notable exception of S02 and the less well-sampled site S07 (Table 2).

### There Is No Core Community in Pozas Rojas

**FIGURE 4:**
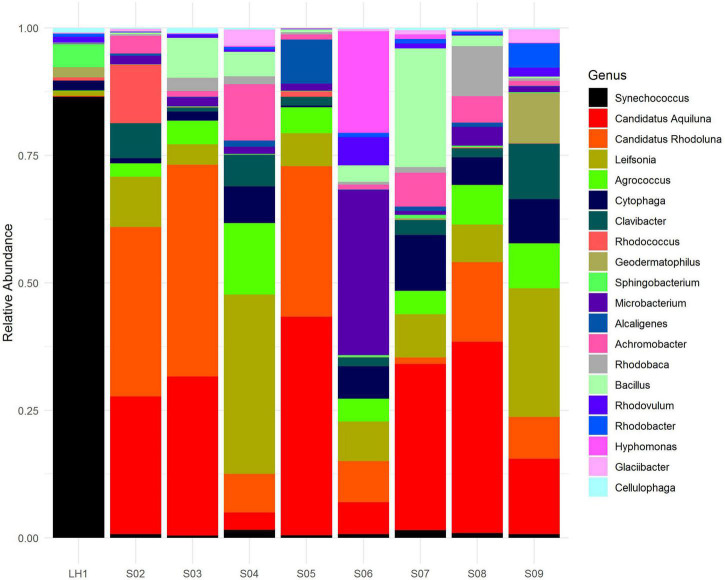
Microbial community composition of the Pozas Rojas system. Relative abundance of 16S reads annotated at the genus level. Only the top 20 most abundant genera are shown in legend.

The divergent community composition of LH (Figure 2) is also evident in the unique versus shared OTUs between Pozas Rojas sites (Supplementary Figure 3), with LH having 1334 unique OTUs, whereas the next site in line, S08, has only 403. Surprisingly, there are no core OTUs that are shared among all sites despite their proximity and the fact that they were a connected water body 2 years prior to this sampling (Supplementary Figure 3). In order to understand such uniqueness, we assigned genera to the most abundant OTUs (>0.1%). With this approach, we were able to identify that 150 genera are shared between the different pozas (Figure 3). On the most divergent site, LH, the marine Cyanobacteria *Synechococcus* is the most abundant, whereas it is rare in the shallower pozas (Figure 4). Meanwhile, most shallow pozas have diverse proportions of the Actinobacteria Candidatus *Rhodoluna*, as well as a high abundance of Rhodobacteraceae along with fewer Cyanobacteria.

### Migration Is Rare Between Los Hundidos and the Pozas

**FIGURE 5:**
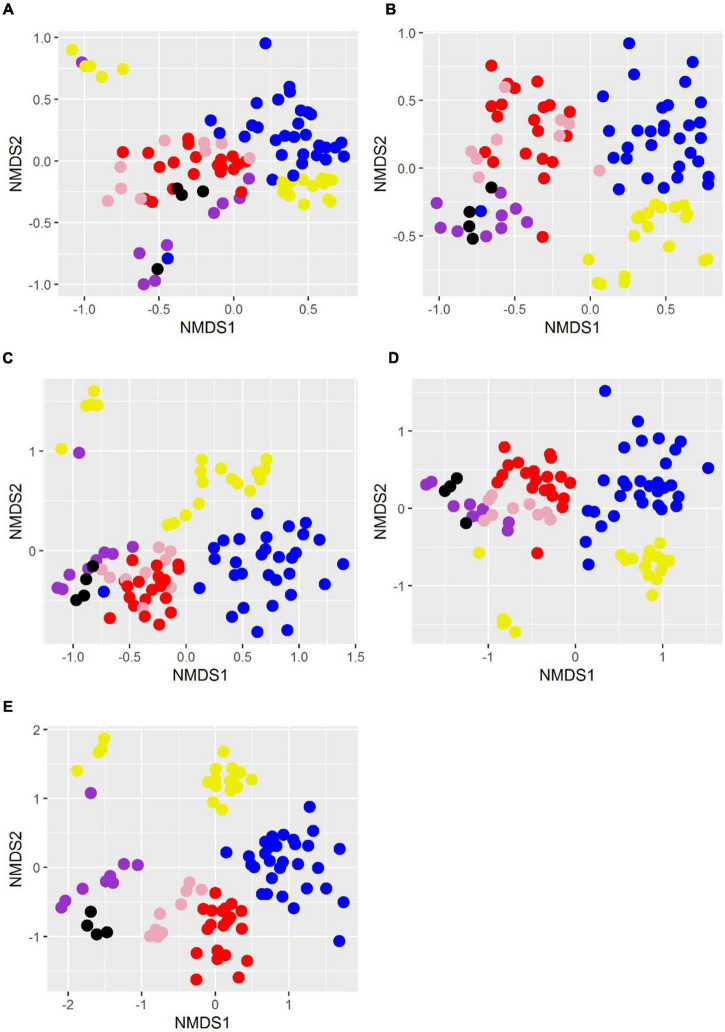
Non-metric multidimensional scaling plots of relative abundances at different taxonomic levels. Taxonomic categories are separated by panels **(A):** phylum, **(B):** class, **(C):** order, **(D):** family, and **(E):** genus. Samples are colored as follows: Pozas Rojas, purple; Lake St. Claire, pink; cold rivers of the world, red; deep water from Iowa, blue; Mediterranean Sea, yellow; Churince, black.

**FIGURE 6:**
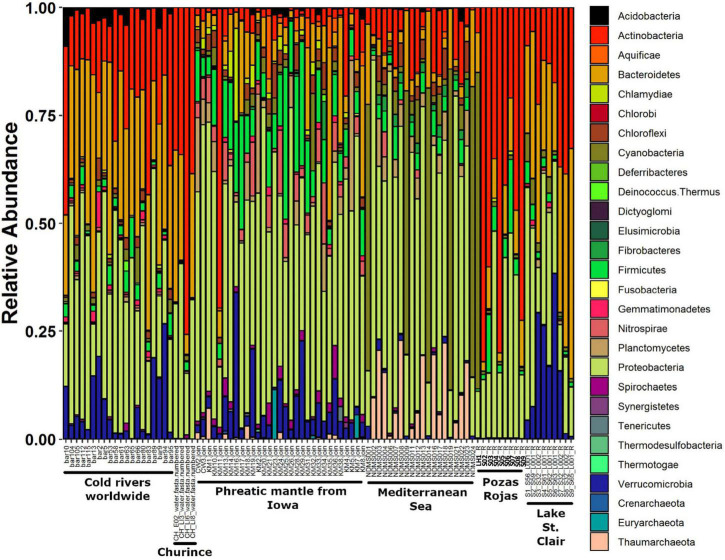
Stacked bar plot of relative abundance of phyla from global samples. Sites to which samples belong are indicated on the *X*-axis.

While at higher taxonomic levels clustering of sites is generally not clear, at the genus level, all Pozas Rojas samples group together and very closely to Churince samples, except for LH, which arranges itself as a part of the epipelagic samples of the Mediterranean Sea (Figure 5). Moreover, a clear difference can be appreciated on the taxonomic profiles of LH1 and the rest of the pozas along similarities with the epipelagic Mediterranean Sea samples at the phylum level, mainly a very high relative abundance of Cyanobacteria followed by Bacteroidetes (Figure 6). This suggests not only that the water source of LH is different from the shallow pozas, but also that superficial migration from that lagoon to the pozas around the lagoon is rare.

## Discussion

All the data show that there is a strong community structure and differentiation between the individual pozas, particularly with the deeper and most stable site, Los Hundidos lagoon. As we stated previously, there are two possibilities: (1) the Grinnellian environmental variables explain most of Pozas Rojas diversity and structure; and (2) each poza is unique because of the Eltonian niche determining community assembly. In this study, we observed a significant differentiation in community structure between pozas given by environmental variables such as temperature and pH. This is to be expected, as these environmental variables are the most important variables in microbial community assembly in general (Hollister et al., 2010; Ren et al., 2015; Yan et al., 2017). However, nutrients and minerals, with the exception of As that is more abundant in pozas S02, S04, and S07, appear to be less relevant variables for community assembly in Pozas Rojas. What is interesting is that LH, environmentally, is part of the large inner cluster (Figure 1); this could be due to the drainage of the hurricane water toward it. Nevertheless, such clustering is not reflected in the microbial community structure, being LH the most divergent site. Therefore, although there is an influence of Grinnellian niche on community assembly, Eltonian processes seem to play a more important role in Pozas Rojas differentiation, potentiated by founder effects on each poza and their further isolation by distance.

Although in this study we are exploring only the water community, the overall diversity in Pozas Rojas is as high as Churince (>5,000 OTUs, Souza et al., 2018). This is remarkable, given the small scale of the study (circa 700 × 500 m) and the subsampling due to the inherent sequencing technical deficiencies. Other aquatic diversities have been reported to be at least an order of magnitude lower, as is the case of Svalbard subglacial ice with 700 OTUs in an equivalent sampling coverage of three glaciers (Perini et al., 2019), or 811 OTUs observed in five sites along the Ganjiang River in China (Wang et al., 2016). It is most interesting that despite the small scale of our study, the richness observed here is double the species obtained in the TARA ocean survey for superficial waters and comparable to the observed in mesopelagic waters at the scale of the world (Sunagawa et al., 2015).

It is possible that the large diversity found in Pozas Rojas is in part the product of a more balanced stoichiometry, reducing the environmental filtering of low phosphorus content typical from CCB (Vázquez-Rosas-Landa et al., 2020). Another possible factor is the pozas differentiation, where a particular founder effect and a cohesive community, the Eltonian niche, isolate each poza from the next one, generating an equitable distribution, as well as a large diversity in most of the pozas. Another role in such diversity and differentiation should be assigned to viruses that we know are particularly diverse (Taboada et al., 2018) as their constant pressure tends to increase diversity as a escape mechanism to predators.

It seems that each poza had a unique process of establishment [a.k.a. founder effect (Peter and Slatkin, 2015)]. In fact, after the homogenization due to the hurricane’s high waters, only pozas S02 and S05 share similar communities (Figure 2), even though they are not so close, nor part of the same environmental cluster (Figure 1). If we take away the lagoon (LH1), most of the unique OTUs go away (1334) (Figure 3). Such a large number of unique OTUs belong mostly to 33 unique genera, with *Synechococcus* being particularly abundant in LH, which is one of the most abundant Cyanobacteria in the ocean along with *Prochlorococcus* (Sunagawa et al., 2015). This result is interesting but not new, as multiple marine lineages such as *Exiguobacterium* (Rebollar et al., 2012), *Bacillus* (Alcaraz et al., 2008; Moreno-Letelier et al., 2012), and marine phages (Desnues et al., 2008) have been previously found on the water systems from Cuatro Cienegas, thus supporting the marine origin of local communities (Souza et al., 2006). However, the observed pronounced differentiation of the pozas from LH may be overestimated, given the acute differences in-depth between the two types of water bodies. It is expected to get a more complete community sampling from a non-stratified shallow poza than a 10-m-deep lagoon from which the sample was taken from the surface. Hence, sampling bias led by the stratification of LH most likely played a role in the observed divergence.

We observe among this large diversity a core of 150 genera (Figure 3), even if there is not a core at the OTU level, suggesting microdiversification processes separating each pond. The shallow ponds are characterized by different proportions of genus from the Microbacteriaceae family with strains related to *Candidatus Rhodoluna*, which has been described to have photorhodopsin that allows for autotrophy; from the same family are the marine genus *Candidatus Aquiluna*, the soil bacteria *Agrococcus*, and the aquatic *Leifsonia* that has been described in continental waters (Reddy et al., 2003; Zhang et al., 2010; Chuon et al., 2021). Beyond the core, at the genus level, 25 genera explain the differences between pozas with a *p* > 0.015 (Supplementary Table 3). It is interesting that most of these bacteria are heterotrophs, but only few of them are primary producers, as the marine Cyanobacteria *Acaryochloris*, an halophile that has low light chlorophyll; *Aphanothece* and *Prochlorothrix*, common in freshwater systems; *Leptolyngbya*, found in the Arctic Ocean; and Chlorobaculum, a green sulfur bacteria with an anoxygenic phototrophic metabolism. The chemoautotroph *Acidithiomicrobium*, an Fe-oxidizing acidophile and thermophile, was also found to be important in the differentiation of the pozas, and as only few species have been described (Norris et al., 2011), it is possible that this one in Pozas Rojas is a new species. Some genera are typical from deep aquifers or marine environments, whereas others are typical from freshwater and soil, therefore representing potential migrants from the local environments that got “dragged” along the flood water to the Pozas Rojas system. Interestingly, such migration did not seem to occur to the larger lagoon.

When comparing the Pozas Rojas system with other water systems of the world that have comparable data in MG-RAST, we observe that each site has a “signature” even at the phylum level determined mostly by their most abundant groups (Figure 6). In this case, Churince and Pozas Rojas appear similar to the cold rivers of the world and phreatic mantle from Iowa, meaning, continental waters. On the other hand, LH from Pozas Rojas is more similar to the epipelagic samples from the Mediterranean Sea, in particular, on their high abundance of Cyanobacteria (Figure 6). This similarity is maintained in the NMDS plot at the phylum, order, and genus taxonomic levels only with the epipelagic fraction of the Mediterranean Sea, which is the zone with the highest abundance of Cyanobacteria in the ocean (Figure 5).

Even though more in-depth studies are needed in this diverse and heterogeneous site, it is captivating that the deep aquifer possibly connected to the deep lagoon is related to the ocean, and its community structure has persisted for at least 30 million years that the highland of central Mexico along with the Sierra Madre Oriental uplifted isolating CCB from the Western Seaway, confirming that CCB is indeed a dynamic lost world where ancestral residents mingle with more recent migrants without losing its particular diverse marine signature. In the last years, efforts toward saving this amazing wetland have yielded fruit as 40 Ha of the wetland is in recovery as two channels that diverted water for irrigation have been partially closed, giving us the luxury of a little more time to understand the dynamics of this unique deep aquifer.

## Data Availability Statement

The datasets presented in this study can be found in online repositories. The names of the repository/repositories and accession numbers can be found below: NCBI:PRJNA785576, MG-RAST:mgp94066.

## Author Contributions

MG-U: initial drafting, data analysis, figure generation, discussion, literature review, and draft correction. VS and LEE: project coordination, discussion, and draft correction. DE-H: raw data processing, discussion, and literature review. JS-P and MV: initial drafting, data analysis, figure generation, discussion, and literature review. LE-A: project coordination, data analysis, discussion, and draft correction. MM-R: data analysis, figure generation, discussion, and literature review. MN-M, JR-P, and CM-G: data analysis, discussion, and literature review. DM-T: data analysis, discussion, literature review, and draft correction. MR-B: DNA extraction and raw data processing. MV-R-L: raw data processing, data analysis, and discussion. All authors contributed to the article and approved the submitted version.

## Conflict of Interest

The authors declare that the research was conducted in the absence of any commercial or financial relationships that could be construed as a potential conflict of interest.

## Publisher’s Note

All claims expressed in this article are solely those of the authors and do not necessarily represent those of their affiliated organizations, or those of the publisher, the editors and the reviewers. Any product that may be evaluated in this article, or claim that may be made by its manufacturer, is not guaranteed or endorsed by the publisher.

## Footnotes

1
http://cran.r-project.org

2
mg-rast.org
