# Supplementary material for: Recent Differentiation of Aquatic Bacterial Communities in a Hydrological System in the Cuatro Ciénegas Basin, After a Natural Perturbation
Source: Front Microbiol. 2022 Apr 28;13:825167. doi: 10.3389/fmicb.2022.825167 (PMC9097865; doi:10.3389/fmicb.2022.825167)
Supplement: Supplementary file 1 [file Data_Sheet_1.docx]

**Supplementary Material**

**Supplementary Figures**

**
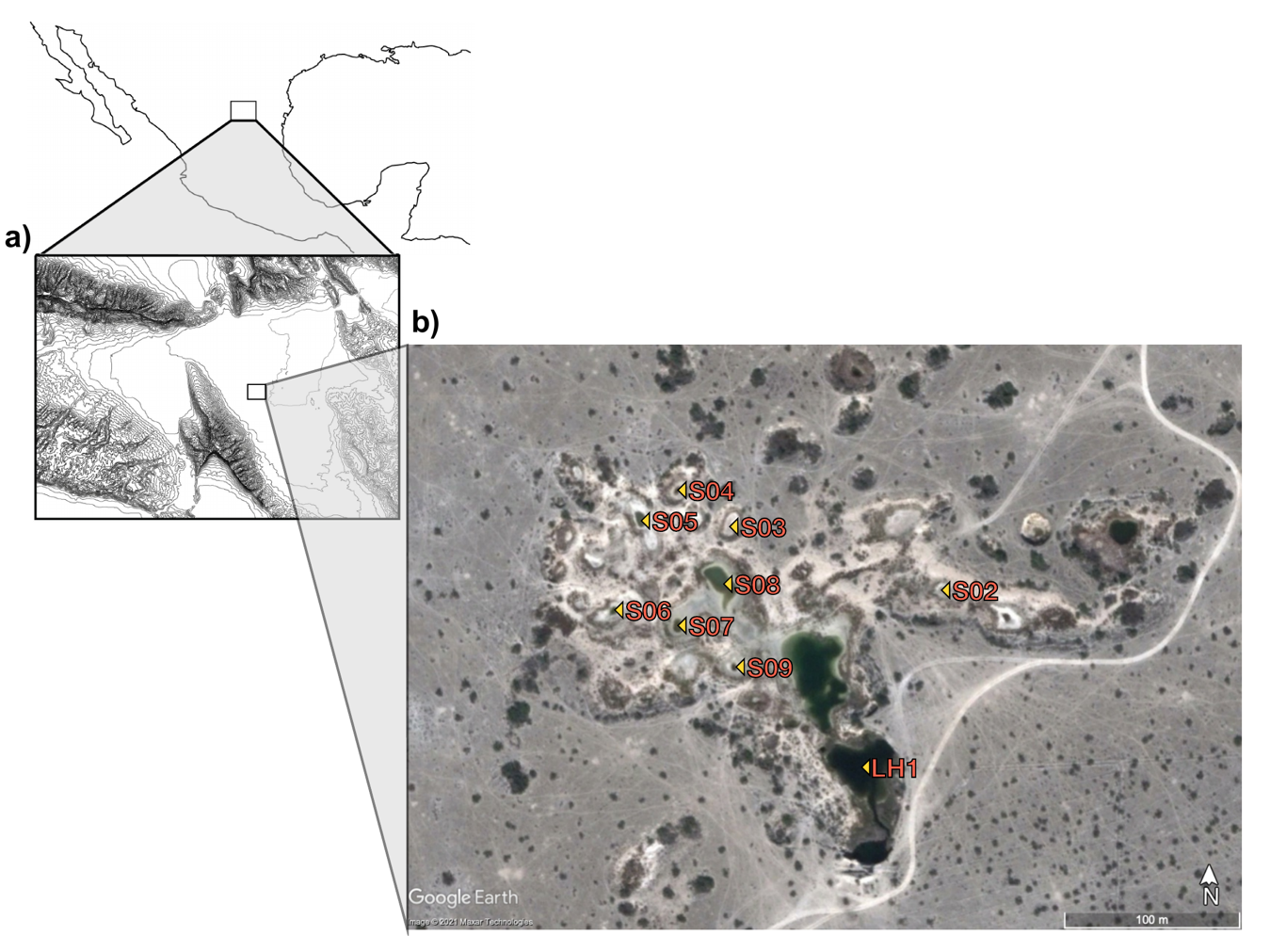
**

**Fig S1. Sample site overview. a)** the Cuatro Ciénegas Basin, where the Pozas Rojas system is located on the eastern side of the valley, and **b)** ponds and lagoons within the Pozas Rojas system (satellite image from 2012), where ponds are designated as S02-S09 and Los Hundidos Lagoon as LH1. Panel **a)** was built using the public sources CONANP 664 (<http://sig.conanp.gob.mx/website/pagsig/mapas_serie.htm>) and CONABIO SNIB 665 (<http://www.conabio.gob.mx/informacion/gis/>). Image on panel b) was retrieved from Google Earth Pro v7.3.4, coordinates 26°52'15.03"N, 102° 1'14.21"O, elevation of 712 m, 2012.


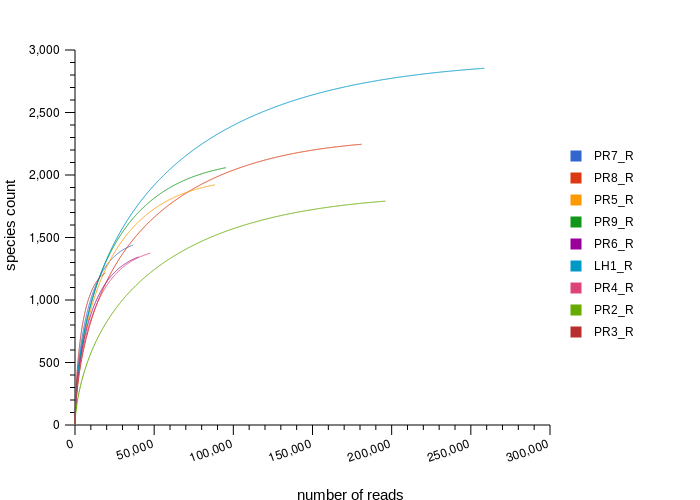


**Fig S2. Rarefaction curves for the Pozas Rojas system.** In this hydrological system, Los Hundidos (LH) lagoon along with ponds S02, S08 and S09 are well sampled in terms of microbial diversity.


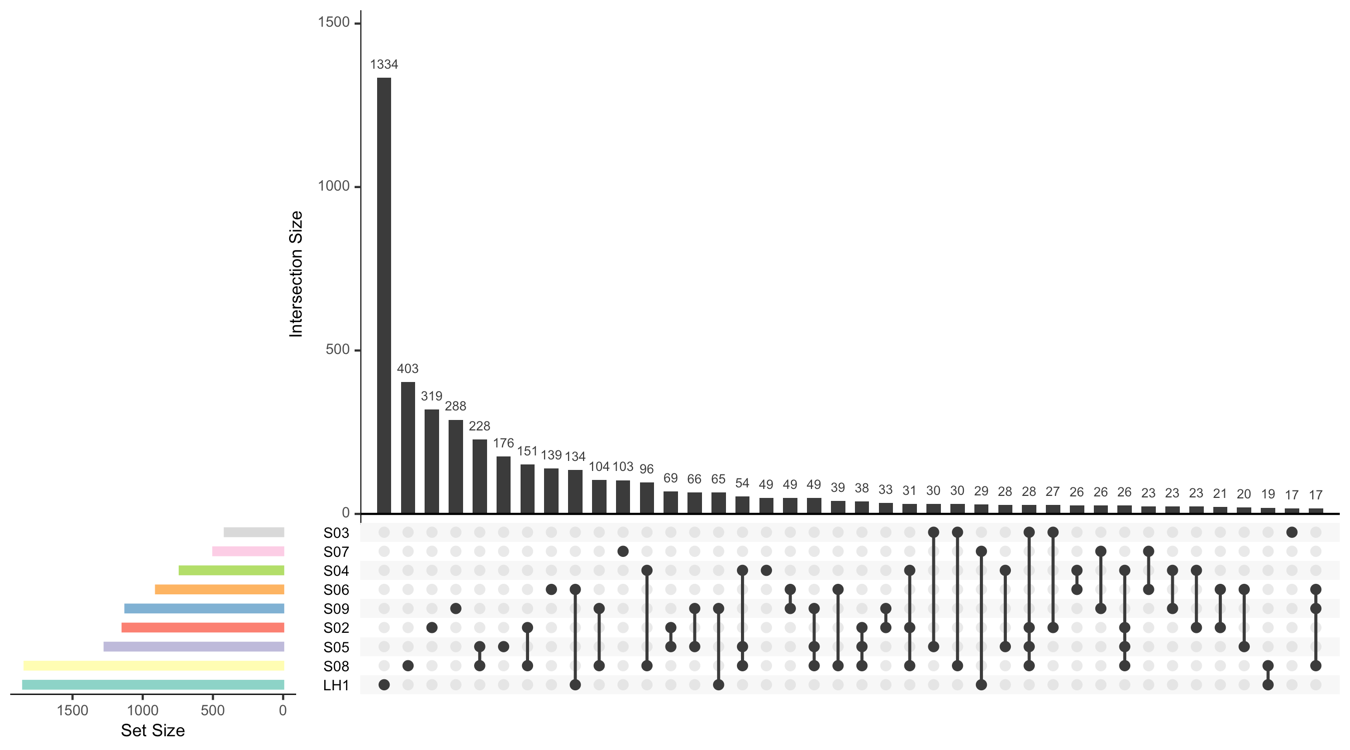


**Fig S3. Upset plot of shared OTUs between pozas.** Connected dots display the intersection of shared genera between each sample. Based on OTUs, there is no core community between all samples. Total number of OTUs in each pond are shown by the horizontal frequency bars.

**Fig S4. Rarefaction curves for hydrological systems around the world. a)** Cold rivers of the world**, b)** Lake St. Clair, **c)** Mediterranean Sea, **d)** Phreatic mantle from Iowa, **e)** Churince (also in Cuatro Ciénegas)

**Supplementary Tables**

**Table S1. Geographic distance from each waterbody from Pozas Rojas system.**

|  | **LH1** | **S02** | **S03** | **S04** | **S05** | **S06** | **S07** | **S08** | **S09** |
| --- | --- | --- | --- | --- | --- | --- | --- | --- | --- |
| **LH1** | 0 | 0,096183236 | 0,113674557 | 0,14421779 | 0,146585725 | 0,132165919 | 0,094369706 | 0,092923418 | 0,05265774 |
| **S02** | 0,096183236 | 0 | 0,126624574 | 0,161489778 | 0,178511334 | 0,190320943 | 0,153386226 | 0,130005959 | 0,121979681 |
| **S03** | 0,113674557 | 0,126624574 | 0 | 0,035468942 | 0,053134962 | 0,082425763 | 0,062764013 | 0,031375827 | 0,074951333 |
| **S04** | 0,14421779 | 0,161489778 | 0,035468942 | 0 | 0,026767334 | 0,072883418 | 0,071579195 | 0,052307566 | 0,098801777 |
| **S05** | 0,146585725 | 0,178511334 | 0,053134962 | 0,026767334 | 0 | 0,049658219 | 0,060487325 | 0,054797331 | 0,096364732 |
| **S06** | 0,132165919 | 0,190320943 | 0,082425763 | 0,072883418 | 0,049658219 | 0 | 0,038908939 | 0,06213286 | 0,079921632 |
| **S07** | 0,094369706 | 0,153386226 | 0,062764013 | 0,071579195 | 0,060487325 | 0,038908939 | 0 | 0,033140474 | 0,041724681 |
| **S08** | 0,092923418 | 0,130005959 | 0,031375827 | 0,052307566 | 0,054797331 | 0,06213286 | 0,033140474 | 0 | 0,046735597 |
| **S09** | 0,05265774 | 0,121979681 | 0,074951333 | 0,098801777 | 0,096364732 | 0,079921632 | 0,041724681 | 0,046735597 | 0 |

**Table S2. Environmental variables for each waterbody from Pozas Rojas System.**

|  | **Temp. (°C)** | **COT (mg/L)** | **NT (mg/L)** | **PT (mg/L)** | **C:N** | **C:P** | **N:P** | **pH** | **Cndct (uS/cm)** | **CO_3_^2-^ (mg/L)** | **HCO_3_^-^ (mg/L)** | **SO_4_^2-^ (mg/L)** | **Cl^-^ (mg/L)** | **Na^+^ (mg/L)** | **K^+^ (mg/L)** | **Ca^2+^ (mg/L)** | **Mg^2+^ (mg/L)** | **Cd (mg/L)** | **Pb (mg/L)** | **As (mg/L)** |
| --- | --- | --- | --- | --- | --- | --- | --- | --- | --- | --- | --- | --- | --- | --- | --- | --- | --- | --- | --- | --- |
| **LH1** | 16.72 | 30.3 | 0.44 | 1.14 | 69.5 | 26.67 | 0.38 | 8.24 | 8660 | 32.43 | 161.86 | 5088 | 742 | 998 | 54 | 636.27 | 671.42 | 0.05 | 0.19 | 0.0242 |
| **S02** | 15.72 | 448.73 | 16.16 | 1.38 | 27.77 | 325.17 | 11.71 | 8.71 | 37600 | 253.56 | 347.71 | 25506 | 5610 | 7330 | 415 | 641.28 | 4253.37 | 0.3 | 1.22 | 0.0986 |
| **S03** | 16.3 | 480.71 | 10.17 | 0.98 | 47.27 | 490.52 | 10.38 | 8.58 | 46200 | 330.22 | 587.52 | 33490 | 7730 | 9563 | 537.5 | 686.37 | 6419.55 | 0.44 | 1.32 | 0.0488 |
| **S04** | 16.58 | 186.23 | 5.08 | 1.15 | 36.66 | 161.94 | 4.42 | 8.64 | 25100 | 159.21 | 341.72 | 15956 | 3060 | 4200 | 253.3 | 621.24 | 2515.56 | 0.19 | 0.64 | 0.103 |
| **S05** | 16.3 | 158.38 | 1.63 | 0.9 | 97.05 | 175.98 | 1.81 | 8.54 | 25900 | 141.52 | 311.74 | 16225 | 3325 | 4325 | 256.8 | 601.2 | 2612.78 | 0.19 | 0.7 | 0.06 |
| **S06** | 16.37 | 499.55 | 11.7 | 1.27 | 42.7 | 393.97 | 9.23 | 8.47 | 41200 | 247.66 | 599.51 | 28864 | 6840 | 8062.5 | 435 | 686.37 | 4809.35 | 0.31 | 1.06 | 0.073 |
| **S07** | 15.7 | 133.46 | 2.98 | 1.13 | 44.85 | 117.9 | 2.63 | 8.63 | 15930 | 85.5 | 263.78 | 10125 | 1660 | 2478 | 132 | 636.27 | 1455.26 | 0.08 | 0.41 | 0.216 |
| **S08** | 16,66 | 247,09 | 7,42 | 1,10 | 33,31 | 223,81 | 6,72 | 8,63 | 30000 | 138,57 | 329,73 | 19626 | 4205 | 5400 | 285,8 | 601,2 | 3183,95 | 0,28 | 0,76 | 0,071 |
| **S09** | 17 | 109.88 | 2.32 | 1.16 | 47.28 | 94.56 | 2 | 8.67 | 21300 | 109.1 | 221.82 | 12178 | 2700 | 3350 | 171.2 | 621.24 | 2673.55 | 0.14 | 0.51 | 0.043 |

**Table S3. Significant genera between all samples based on a PCoA analysis.** Genera below 0.05 are shown, and plausibly explain most of the compositional variance between each sample.

| **Genus** | **p-value** | **Genus** | **p-value** |
| --- | --- | --- | --- |
| Acaryochloris | 0.01200 | Halobacillus | 0.02700 |
| Acidithiomicrobium | 0.00100 | Hymenobacter | 0.00800 |
| Acidovorax | 0.00700 | Jannaschia | 0.00800 |
| Akkermansia | 0.02000 | Ketogulonicigenium | 0.03900 |
| Alistipes | 0.03600 | Ktedonobacter | 0.01500 |
| Alkalilimnicola | 0.04500 | Laceyella | 0.01600 |
| Aminobacter | 0.02500 | Lactobacillus | 0.00900 |
| Amycolatopsis | 0.01000 | Legionella | 0.01900 |
| Aphanothece | 0.01500 | Leptolyngbya | 0.01800 |
| Azorhizobium | 0.00200 | Maricaulis | 0.02400 |
| Bifidobacterium | 0.03500 | Methylacidiphilum | 0.01700 |
| Blastomonas | 0.02700 | Methylarcula | 0.05000 |
| Bradyrhizobium | 0.02300 | Microscilla | 0.04100 |
| Candidatus_Aquiluna | 0.04300 | Mycoplasma | 0.03100 |
| Cellulosilyticum | 0.02200 | Novispirillum | 0.01500 |
| Chitinophaga | 0.04200 | Oceanobacillus | 0.00500 |
| Chlorobaculum | 0.04000 | Paenibacillus | 0.00700 |
| Finegoldia | 0.00900 | Pelobacter | 0.02000 |
| Flexibacter | 0.01500 | Prochlorothrix | 0.02800 |
| Flexithrix | 0.00500 | Prolixibacter | 0.04800 |
| Geoalkalibacter | 0.02900 | Pseudoalteromonas | 0.03700 |
| Geobacter | 0.00500 | Rhizobium | 0.01100 |
| Robiginitalea | 0.04600 | Synechococcus | 0.03500 |
| Rubritalea | 0.01200 | Synechocystis | 0.03200 |
| Segniliparus | 0.03400 | Terribacillus | 0.00500 |
| Shewanella | 0.00500 | Thermoactinomyces | 0.02100 |
| Sphingobacterium | 0.02400 | Thermoleophilum | 0.03500 |
| Spiroplasma | 0.00600 | Tropheryma | 0.00300 |
| Spirulina | 0.01400 | Tsukamurella | 0.03400 |
| Sporolactobacillus | 0.01100 |  |  |
